# Co-enrolment in critical care trials: a secondary analysis of the RECOVERY-RS trial

**DOI:** 10.1186/s13054-025-05774-0

**Published:** 2025-11-20

**Authors:** Christopher Eleftheriou, Chen Ji, Ranjit Lall, Daniel F. McAuley, Gavin D. Perkins, Keith Couper

**Affiliations:** 1 Warwick Clinical Trials Unit, University of Warwick, Coventry, CV4 7AL UK; 2 Wellcome-Wolfson Institute for Experimental Medicine, School of Medicine, Dentistry & Biomedical Sciences, Queen’s University Belfast, Belfast, UK; 3 Regional Intensive Care Unit, Royal Victoria Hospital, Belfast, UK; 4 Critical Care Unit, University Hospitals Birmingham NHS Foundation Trust, Birmingham, UK

Co-enrolment describes the process by which an individual patient can participate in more than one clinical study at the same time. In critical care, the limited pool of potential patients means that co-enrolment can be an important strategy in ensuring successful trial delivery. Co-enrolment does, however, create some important challenges, including regulatory considerations, participant burden, and the potential influence on study findings [1]. To date, relatively few critical care trials have described co-enrolment practices.

During the COVID-19 pandemic, the urgent need to identify effective treatment strategies drove the rapid development and delivery of clinical studies, potentially increasing opportunities for co-enrolment. We undertook a secondary analysis of the RECOVERY-RS (Randomized Evaluation of COVID-19 Therapy–Respiratory Support) trial to: (i) quantify the incidence of co-enrolment; (ii) evaluate the variability in co-enrolment across hospitals and (iii) explore the influence of co-enrolment on trial findings [2]. 

RECOVERY-RS was a pragmatic three-armed, open label randomised clinical trial that was rapidly set-up during the early phases of the COVID-19 pandemic to evaluate the clinical effectiveness of non-invasive respiratory support strategies in COVID-19 acute hypoxaemic respiratory failure [2]. Patients were randomised to continuous positive airway pressure (CPAP), high-flow nasal oxygen (HFNO), or conventional oxygen therapy (COT). COT was used as the control group for comparisons with both CPAP and HFNO. RECOVERY-RS was prospectively registered (ISRCTN16912075) and received ethical approval from the London - Brighton & Sussex Research Ethics Committee (20/HRA/1696). This secondary analysis was approved by the University of Warwick Biomedical and Scientific Research Ethics Committee (BSREC 127/23–24) Fig. 1.

We categorised co-enrolment based on the study design of the co-enrolled study, namely interventional or observational. To assess the potential effect of co-enrolment on trial findings, we used logistic regression to estimate the odds ratios (ORs) and 95% confidence intervals (CIs) for the outcomes of tracheal intubation and mortality across three groups: (1) all participants; (2) participants co-enrolled in any study; and (3) participants co-enrolled in an interventional study.

Between April 2020 and May 2021, 1,273 patients were randomised into RECOVERY-RS across 48 hospitals. Co-enrolment occurred in 789 (62.0%) participants (supplemental table S1). Of these, 396 (50.2%) were co-enrolled only into an interventional study, 193 (24.5%) were co-enrolled only into an observational study, and 200 (25.3%) were co-enrolled into both an observational and interventional study (supplemental table S2). Across the 31 hospitals recruiting five or more participants, co-enrolment rates ranged from 25% to 97% (IQR 52%−79%) (supplemental figure S1).

**Fig. 1:**
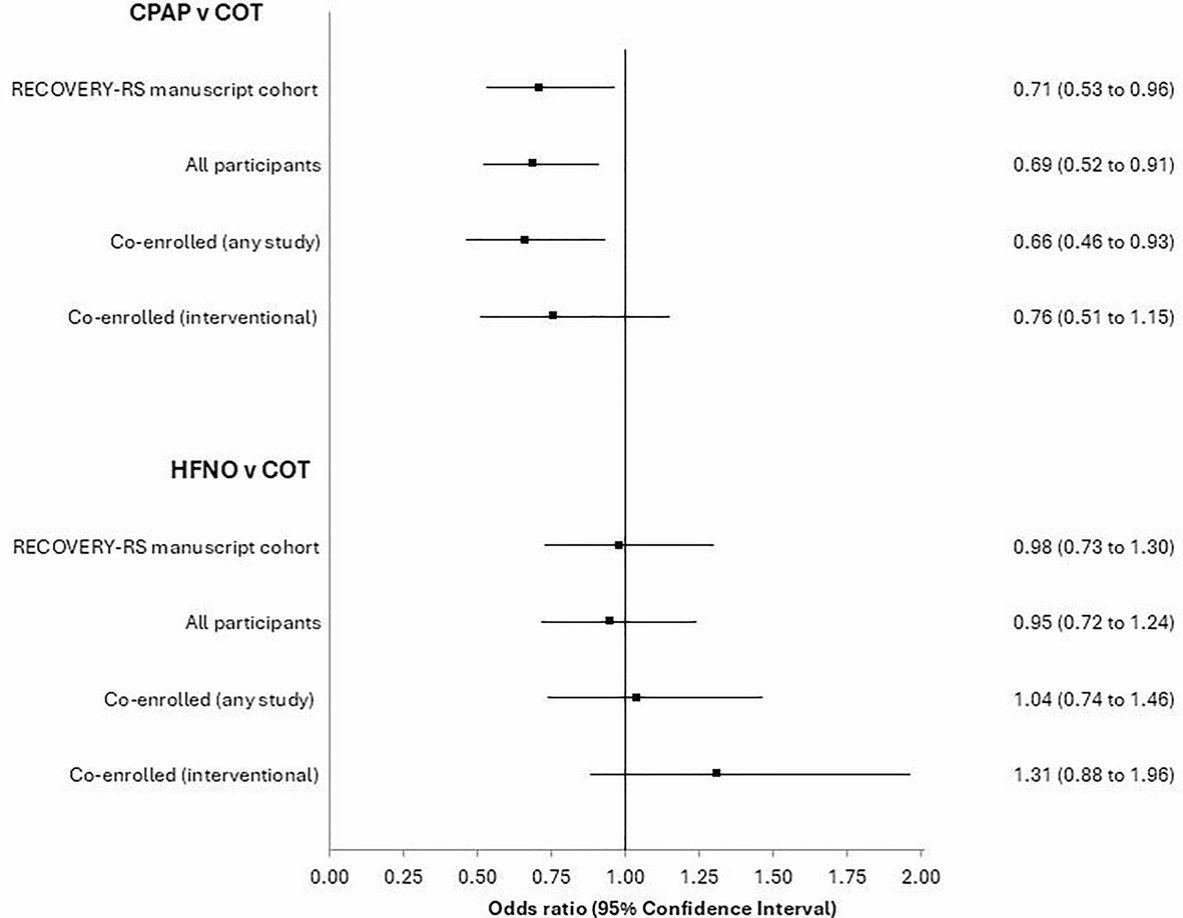
Influence of co-enrolment on study findings- outcome: tracheal intubation. CPAP- Continuous positive airway pressure; COT- Conventional oxygen therapy; HFNO- High-flow nasal oxygen

Co-enrolled participants, compared with participants who were not co-enrolled, were more often male (69% v 62%, *p* = 0.007), of white ethnicity (74% v 67%, *p* = 0.006), and had no co-morbidities (40% v 33%, *p* = 0.013) (supplemental table S1). Co-enrolled participants seemed to be more severely unwell, as reflected by a lower baseline SpO_2_:FiO_2_ and PaO_2_:FiO_2_ ratio and increased use of awake prone positioning. Admission to critical care was more common in co-enrolled participants (62% v 56%, *p* = 0.022), but there was no statistically significant difference in the rate of tracheal intubation (41% v 36%, *p* = 0.103) or mortality (18 v 20%, *p* = 0.361).

Across our analyses of different patient cohorts (all patients, patients co-enrolled into either an observational or interventional study; patients co-enrolled into another interventional study) for the outcome of tracheal intubation, we observed some changes in reported odds ratio across groups, particularly for the comparison of HFNO and conventional oxygen therapy (figure one; supplemental table S3-S4). However, limiting these analyses to these specific participant cohorts did not materially influence our interpretation of the study findings for the outcomes of either tracheal intubation or mortality (figure one; supplemental table S3-S4).

Our findings show that high rates of co-enrolment in critical care trials are achievable in specific contexts. Previous reports of co-enrolment in critical care trials have typically reported co-enrolment rates of approximately 20%−40% [3, 4]. The high rate of co-enrolment observed in RECOVERY-RS was likely driven by the unique study context, particularly a small number of research studies targeting overlapping populations, the high number of hospitalised patients with COVID-19, and the strong commitment of staff and patients across UK hospitals to deliver COVID-19 research. Our findings provide reassurance that co-enrolment may have little influence on study findings. There is an opportunity for novel research designs, such as the Confederation of Respiratory Critical Care Trials, to provide new insights into co-enrolment through the collection of co-enrolment data across trials and integration of these data in study analyses [5]. 

In this secondary analysis of the RECOVERY-RS trial, 62% patients were co-enrolled in another study, although rates of co-enrolment varied markedly across hospitals. Whilst there were important differences between co-enrolled and non-co-enrolled patients, co-enrolment did not materially influence trial findings.

## Supplementary Information

Supplementary Material 1.

## Data Availability

Due to study approvals, relevant datasets are not publicly available, but deidentified patient-level data from the RECOVERY-RS trial may be made available for approved research on application to the RECOVERY-RS chief investigators (Professor Gavin Perkins and Professor Daniel F McAuley).

## Abbreviations

COT: Conventional oxygen therapy
CPAP: Continuous positive airway pressure
HFNO: High-flow nasal oxygen
RECOVERY-RS: Randomized Evaluation of COVID-19 Therapy-Respiratory Support
