# Supplementary material for: Co-enrolment in critical care trials: a secondary analysis of the RECOVERY-RS trial
Source: Crit Care. 2025 Nov 20;29:499. doi: 10.1186/s13054-025-05774-0 (PMC12636142; doi:10.1186/s13054-025-05774-0)
Supplement: Supplementary file 1 — Supplementary Material 1. [file 13054_2025_5774_MOESM1_ESM.docx]

**Co-enrolment in critical care trials: a secondary analysis of the Recovery-RS trial**

**Supplementary materials**

Contents

[Table S1: Comparison of co-enrolled participants with non-co-enrolled participants 2](#_Toc213141336)

[Table S2: Summary of co-enrolment by study type (interventional and observational) 4](#_Toc213141337)

[Table S3: Influence of co-enrolment on study findings- comparison: continuous positive airway pressure v conventional oxygen therapy 5](#_Toc213141338)

[Table S4: Influence of co-enrolment on study findings- comparison: high-flow nasal oxygen v conventional oxygen therapy 6](#_Toc213141339)

[Figure S1: Percentage of patients co-enrolled by hospitals recruiting at least five patients 7](#_Toc213141340)

# Table S1: Comparison of co-enrolled participants with non-co-enrolled participants

|  |  | All patients  (n=1273) | Patient co-enrolled  (n=789) | Patient not co-enrolled (n=484) | p-value |
| --- | --- | --- | --- | --- | --- |
|  |  |  |  |  |  |
| Age- mean (SD) | | 57.4 (12.7) | 57.1 (12.1) | 57.8 (13.7) | 0.397 |
| Sex- male- n(%) | | 844 (66.3%) | 545 (69.1%) | 299 (61.8%) | 0.007 |
| Ethnicity- n(%) | |  |  |  |  |
|  | Black, Asian and minority ethnic | 334 (28.7%) | 189 (25.9%) | 145 (33.4%) | 0.006 |
|  | White | 831 (71.3%) | 542 (74.1%) | 289 (66.6%) |  |
| Clinical frailty score- n(%)† | |  |  |  |  |
|  | CFS1-3: Very fit to managing well) | 1157 (92.6%) | 729 (93.6%) | 428 (90.9%) | 0.077 |
|  | CFS4-9: very mild frailty to terminally ill) | 93 (7.4%) | 50 (6.4%) | 43 (9.1%) |  |
| Past medical history- n(%) | |  |  |  |  |
|  | No co-morbidity | 476(37.7%) | 317 (40.3%) | 159 (33.3%) | 0.013 |
|  | End stage renal failure requiring renal replacement therapy | 13 (1.0%) | 6 (0.8%) | 7 (1.5%) | 0.232 |
|  | Congestive cardiac failure | 11 (0.9%) | 6 (0.8%) | 5 (1.0%) | 0.601 |
|  | Chronic lung disease | 183 (14.5%) | 116 (14.7%) | 67 (14.0%) | 0.712 |
|  | Coronary heart disease | 104 (8.2%) | 63 (8.0%) | 41 (8.6%) | 0.728 |
|  | Dementia | 8 (0.6%) | 4 (0.5%) | 4 (0.8%) | 0.477 |
|  | Diabetes requiring medication | 275 (21.7%) | 170 (21.6%) | 105 (21.9%) | 0.894 |
|  | Hypertension | 448 (35.4%) | 266 (33.8%) | 182 (38.0%) | 0.126 |
|  | Uncontrolled or active malignancy | 24 (1.9%) | 11 (1.4%) | 13 (2.7%) | 0.096 |
|  | Morbid obesity‡ | 218 (17.2%) | 136 (17.3%) | 82 (17.2%) | 0.967 |
| COVID-19 infection status- n(%) | |  |  |  |  |
|  | Confirmed | 1090 (85.9%) | 689 (87.3%) | 401 (83.5%) | 0.06 |
|  | Suspected | 179 (14.1%) | 100 (12.7%) | 79 (16.5%) |  |
| Time from symptom onset to admission (days)- median (IQR) | | 7.0 (5.0-10.0) | 8.0 (6.0-10.0) | 7.0 (4.0-10.0) | 0.027 |
| Time from symptom onset to randomisation (days)- median (IQR) | | 9.0 (7.0-12.0) | 9.0 (7.0-12.0) | 9.0 (6.0-12.0) | 0.039 |
| Treatment period- n(%)* | |  |  |  |  |
|  | Pre-July 2020 | 138 (10.8%) | 103 (13.1%) | 35 (7.2%) | <0.001 |
|  | July 2020-January 2021 | 882 (69.3%) | 548 (69.5%) | 334 (69.0%) |  |
|  | Post-January 2021 | 253 (19.9%) | 138 (17.5%) | 115 (23.8%) |  |
| Baseline physiology- median (IQR) | |  |  |  |  |
|  | FiO_2_ | 0.6 (0.40-0.80) | 0.60 (0.40-0.85) | 0.60 (0.40-0.80) | 0.002 |
|  | SpO_2_ (%) | 93 (91-95) | 93 (91-95) | 94 (92-95) | 0.675 |
|  | PaO_2_ (kPa) | 8.9 (7.9-10.2) | 8.9 (7.8-10.2) | 9.1 (7.9-10.2) | 0.306 |
|  | PaCO_2_ (kPa) | 4.4 (4.0-4.9) | 4.4 (4.0-4.8) | 4.4 (4.0-4.9) | 0.428 |
|  | SpO_2_:FiO_2_ ratio | 156.7 (113.8-232.5) | 155.0 (110.0-230.0) | 156.7 (117.5-235.0) | 0.001 |
|  | PaO_2_:FiO_2_ ratio | 15.20 (10.9-21.3) | 14.49 (10.61-20.00) | 16.17 (11.86-23.50) | <0.001 |
|  | Respiratory rate (breaths/minute) | 24 (20-29) | 24 (20-29) | 24 (20-30) | 0.614 |
| Treatment allocation- n (%) | |  |  |  |  |
|  | CPAP | 380 (29.9%) | 259 (32.8%) | 121 (25.0%) | 0.012 |
|  | HFNO | 418 (32.8%) | 250 (31.7%) | 168 (34.7%) |  |
|  | COT | 475 (37.3%) | 280 (35.5%) | 195 (40.3%) |  |
| Treatment duration (hours)- median (IQR) | | 58.4 (24.0-110.8) | 60.5 (24.0-110.1) | 52.8 (24.0-115.0) | 0.905 |
| Received awake prone position- n (%) | | 702 (67.4%) | 487 (71.4%) | 215 (59.7%) | <0.001 |
| Clinical outcomes | |  |  |  |  |
|  | Admitted to critical care- n(%) | 737 (59.6%) | 483 (62.1%) | 254 (55.5%) | 0.022 |
|  | Critical care length of stay (days)- median (IQR) | 11.0 (5.3-21.8) | 12.0 (5.6-24.0) | 9.4 (4.9-18.1) | 0.003 |
|  | Tracheal intubation within 30-days- n(%) | 494 (39.2%) | 323 (40.9%) | 171 (36.3%) | 0.103 |
|  | Intubation duration (days)- median (IQR) | 14.0 (7.0-25.0) | 16.0 (7.8-26.0) | 11.0 (5.0-20.0) | 0.005 |
|  | Mortality in 30-days- n(%) | 231 (18.3%) | 138 (17.5%) | 93 (19.5%) | 0.361 |
|  | Hospital length of stay (days)- median (IQR) | 11.0 (7.0-20.0) | 12.0 (7.0-22.0) | 10.0 (6.0-18.0) | <0.001 |
| †- Clinical frailty score is measured on a 9-point scale, ranging from one (very fit) to 9 (terminally ill)  ‡- Morbid obesity was defined as body mass index greater than 35 (kilograms/metre^2^)  *- Treatment phases were defined based on the introduction of dexamethasone in to standard care in July 2020 and introduction of tocilizumab in to standard care in January 2021  Abbreviations  CFS- Clinical Frailty Score; COT- Conventional oxygen therapy; CPAP- Continuous positive airway pressure; HFNO- High-flow nasal oxygen; IQR- Interquartile range; SD- standard deviation.  P-values are based on the comparison of co-enrolled and non-co-enrolled patients. For categorical data, groups are compared using a chi-squared test. Normally distributed continuous data are reported as mean (standard deviation) and groups compared using an independent t-test. Non-normally distributed continuous data are reported as median (interquartile range) and compared using a Mann-Whitney U test. | | | | | |

# Table S2: Summary of co-enrolment by study type (interventional and observational)

|  |  |  | Patients  (n=1273) |
| --- | --- | --- | --- |
| Co-enrolment: Any study type (observational or interventional) | |  |  |
|  | Any co-enrolment- Yes- n(%) |  | 789 (62.0%) |
|  | Number of co-enrolled studies- n(%) |  | (n=789) |
|  |  | 1 | 548 (69.5%) |
|  |  | 2 | 199 (25.2%) |
|  |  | 3 | 39 (4.9%) |
|  |  | 4 | 3 (0.4%) |
| Co-enrolment: observational study | |  |  |
|  | Any co-enrolment to observational study- Yes- n(%) |  | 393 (30.9%) |
|  | Number of co-enrolled observational studies- n(%) |  | (n=393) |
|  |  | 1 | 358 (91.1%) |
|  |  | 2 | 31 (7.9%) |
|  |  | 3 | 4 (1.0%) |
|  |  | 4 | 0 (0%) |
| Co-enrolment: interventional study | |  |  |
|  | Any co-enrolment to interventional study- Yes- n(%) |  | 596 (46.8%) |
|  | Number of co-enrolled interventional studies- n(%) |  | (n=596) |
|  |  | 1 | 550 (92.3%) |
|  |  | 2 | 45 (7.6%) |
|  |  | 3 | 1 (0.2%) |
|  |  | 4 | 0 (0%) |

# Table S3: Influence of co-enrolment on study findings- comparison: continuous positive airway pressure v conventional oxygen therapy

|  |  | CPAP  n/N (%) | Conventional oxygen therapy-  n/N (%) | Unadjusted odds ratio (95% confidence interval |
| --- | --- | --- | --- | --- |
| Outcome: tracheal intubation | |  |  |  |
|  | RECOVERY-RS trial manuscript cohort* | 126/377 (33.4%) | 147/356 (41.3%) | 0.71 (0.53 to 0.96) |
|  | All patients | 126/377 (33.4%) | 198/468 (42.3%) | 0.69 (0.52 to 0.91) |
|  | All co-enrolled patients | 88/259 (34.0%) | 123/280 (43.9%) | 0.66 (0.46 to 0.93) |
|  | Co-enrolled to interventional studies | 64/200 (32.0%) | 78/204 (38.2%) | 0.76 (0.51 to 1.15) |
| Outcome: mortality | |  |  |  |
|  | RECOVERY-RS trial manuscript cohort* | 63/378 (16.7%) | 69/359 (19.2%) | 0.84 (0.58 to 1.23) |
|  | All patients | 63/378 (16.7%) | 90/471 (19.1%) | 0.85 (0.59 to 1.21) |
|  | All co-enrolled patients | 40/259 (15.4%) | 51/280 (18.2%) | 0.82 (0.52 to 1.29) |
|  | Co-enrolled to interventional studies | 30/200 (15.0%) | 34/204 (16.7%) | 0.88 (0.52 to 1.51) |
| *- The analysis in the primary report of RECOVERY-RS was based on intention-to-treat, and individual patients were only included in comparisons to which they could potentially have been randomised. For this analysis, due to the dataset available, we included all conventional oxygen therapy patients in the comparison with continuous positive airway pressure, such that the number of patients is higher than that reported in the RECOVERY-RS trial paper.  CPAP- Continuous positive airway pressure | | | | |

# Table S4: Influence of co-enrolment on study findings- comparison: high-flow nasal oxygen v conventional oxygen therapy

|  |  | High-flow nasal oxygen-  n/N (%) | Conventional oxygen therapy-  n/N (%) | Unadjusted odds ratio (95% confidence interval |
| --- | --- | --- | --- | --- |
| Outcome: tracheal intubation | |  |  |  |
|  | RECOVERY-RS trial manuscript cohort | 170/415 (41.0%) | 153/368 (41.6%) | 0.98 (0.73 to 1.30) |
|  | All patients | 170/415 (41.0%) | 198/468 (42.3%) | 0.95 (0.72 to 1.24) |
|  | All co-enrolled patients | 112/250 (44.8%) | 123/280 (43.9%) | 1.04 (0.74 to 1.46) |
|  | Co-enrolled to interventional studies | 86/192 (44.8%) | 78/204 (38.2%) | 1.31 (0.88 to 1.96) |
| Outcome: mortality | |  |  |  |
|  | RECOVERY-RS trial manuscript cohort | 78/416 (18.8%) | 74/370 (20.0%) | 0.92 (0.65 to 1.32) |
|  | All patients | 78/416 (18.8%) | 90/471 (19.1%) | 0.98 (0.70 to 1.37) |
|  | All co-enrolled patients | 47/250 (18.8%) | 51/280 (18.2%) | 1.04 (0.67 to 1.61) |
|  | Co-enrolled to interventional studies | 36/192 (18.8%) | 34/204 (16.7%) | 1.15 (0.69 to 1.93) |
| *- The analysis in the primary report of RECOVERY-RS was based on intention-to-treat, and individual patients were only included in comparisons to which they could potentially have been randomised. For this analysis, due to the dataset available, we included all conventional oxygen therapy patients in the comparison with high-flow nasal oxygen, such that the number of patients is higher than that reported in the RECOVERY-RS trial paper. | | | | |

# Figure S1: Percentage of patients co-enrolled by hospitals recruiting at least five patients


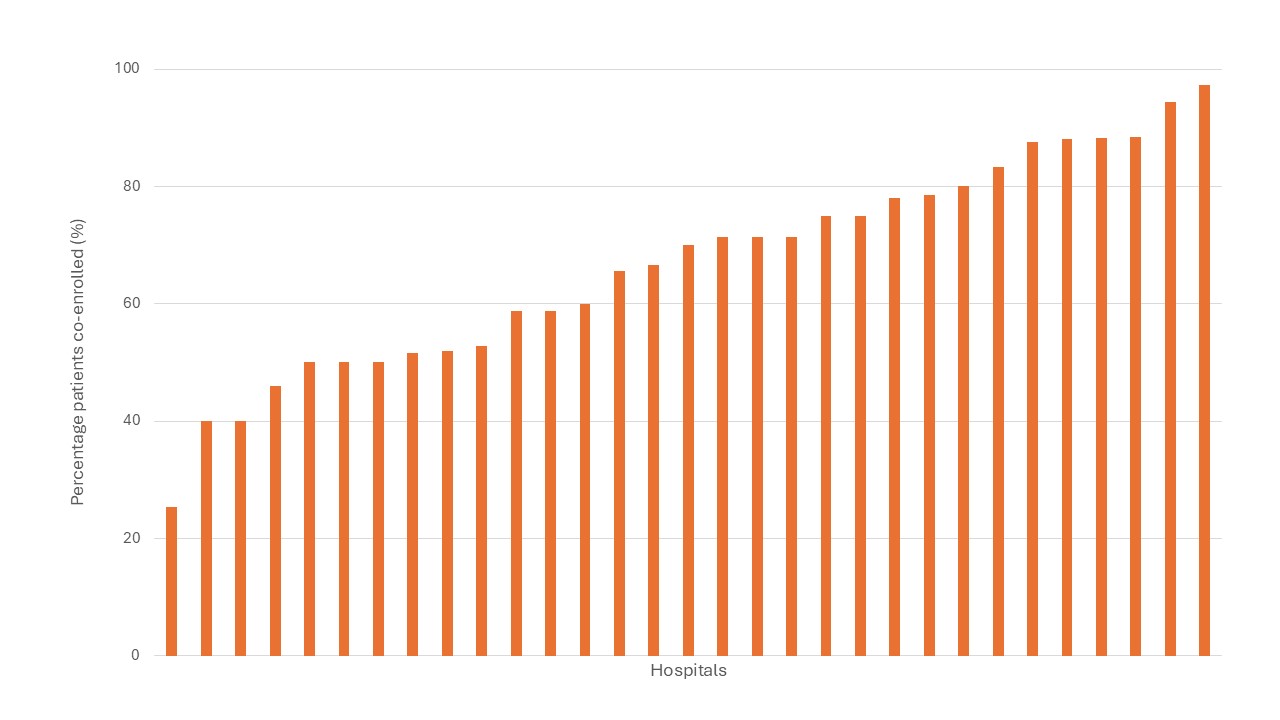


Each bar represents one hospital
